# Characterization of Silk Fibroin Modified Surface: A Proteomic View of Cellular Response Proteins Induced by Biomaterials

**DOI:** 10.1155/2014/209469

**Published:** 2014-03-25

**Authors:** Ming-Hui Yang, Shyng-Shiou Yuan, Tze-Wen Chung, Shiang-Bin Jong, Chi-Yu Lu, Wan-Chi Tsai, Wen-Cheng Chen, Po-Chiao Lin, Pei-Wen Chiang, Yu-Chang Tyan

**Affiliations:** ^1^Instrument Technology Research Center, National Applied Research Laboratories, Hsinchu 300, Taiwan; ^2^Department of Medical Research, Kaohsiung Medical University Chung-Ho Memorial Hospital, Kaohsiung 807, Taiwan; ^3^Translational Research Center, Kaohsiung Medical University Chung-Ho Memorial Hospital, Kaohsiung 807, Taiwan; ^4^Department of Obstetrics and Gynecology, Kaohsiung Medical University Chung-Ho Memorial Hospital, Kaohsiung 807, Taiwan; ^5^School of Medicine, College of Medicine, Kaohsiung Medical University, Kaohsiung 807, Taiwan; ^6^Department of Biomedical Engineering, National Yang-Ming University, Taipei 112, Taiwan; ^7^Department of Medical Imaging and Radiological Sciences, Kaohsiung Medical University, Kaohsiung 807, Taiwan; ^8^Department of Nuclear Medicine, Kaohsiung Medical University Chung-Ho Memorial Hospital, Kaohsiung 807, Taiwan; ^9^Department of Biochemistry, College of Medicine, Kaohsiung Medical University, Kaohsiung 807, Taiwan; ^10^National Sun Yat-Sen University-Kaohsiung Medical University Joint Research Center, Kaohsiung 804, Taiwan; ^11^Department of Medical Laboratory Science and Biotechnology, Kaohsiung Medical University, Kaohsiung 807, Taiwan; ^12^Department of Laboratory Medicine, Kaohsiung Medical University Hospital, Kaohsiung 807, Taiwan; ^13^Department of Fiber and Composite Materials, College of Engineering, Feng Chia University, Taichung 407, Taiwan; ^14^Department of Chemistry, National Sun Yat-Sen University, Kaohsiung 804, Taiwan; ^15^Center of Biomedical Engineering and System Biology, Kaohsiung Medical University, Kaohsiung 807, Taiwan

## Abstract

The purpose of this study was to develop the pathway of silk fibroin (SF) biopolymer surface induced cell membrane protein activation. Fibroblasts were used as an experimental model to evaluate the responses of cellular proteins induced by biopolymer material using a mass spectrometry-based profiling system. The surface was covered by multiwalled carbon nanotubes (CNTs) and SF to increase the surface area, enhance the adhesion of biopolymer, and promote the rate of cell proliferation. The amount of adhered fibroblasts on CNTs/SF electrodes of quartz crystal microbalance (QCM) greatly exceeded those on other surfaces. Moreover, analyzing differential protein expressions of adhered fibroblasts on the biopolymer surface by proteomic approaches indicated that CD44 may be a key protein. Through this study, utilization of mass spectrometry-based proteomics in evaluation of cell adhesion on biopolymer was proposed.

## 1. Introduction

Biomaterials play important roles in regenerative medicine, tissue engineering, and drug delivery [1]. The construction of engineered scaffolds or matrices with chemical and physical surface properties that enable them to interact favorably with cells is important [2]. Cell proliferation, differentiation, and regeneration of tissues all depend upon the interactions between biomaterial surfaces and cells. For the responses of cells to biomaterials, both a cell-count method of counting nuclei stains and the MTT (3-(4,5-cimethylthiazol-2-yl)-2,5-diphenyl tetrazolium bromide) or BrdU (5-bromo-2′-deoxyuridine) assays are less accurate than usual studies, because small parts of cells adhere onto the biomaterial surfaces. Recently, using molecular expression-based methods such as flow cytometric analysis, immunofluorescent labeling and immunoblotting of cells were developed for determining the responses at cellular levels in cell adhesion onto biomaterials [3]. In addition, the identification of proteins that are involved must be known to enable the assays to be properly carried out. The advantage of using the proteomic approach is that new proteins that influence the interactions of cells and biomaterials may be found.

Multiwalled carbon nanotubes (CNTs) have a large surface area and have been extensively studied for a variety of purposes, such as sensors, fuel cells, and device patterning [4, 5]. CNTs have good biocompatibility with cells and support cellular behavior proliferation as well as differentiation of cells in the presence of induction medium. Additionally CNTs substrates show good cell viability, spreading, and physical adhesion. Silk fibroin (SF) is a protein with bulky hydrophobic domains [6] and can be easily purified as sericin-free silk-based biomaterials. Such material is highly applicable due to its low immune response characteristics. SF-based biomaterials have been investigated in the form of files, fibers, hydrogels, particles, and scaffolds [7–10] and in applications of vascular, neural, skin, bone, and cartilage tissue regeneration [11–14]. Increasingly, SF is exploited in other areas of biomedical science, as a result of new knowledge of its processing and properties like mechanical strength, elasticity, biocompatibility, and controllable biodegradability [15]. These properties of SF are particularly useful for tissue engineering.

“Proteome” and “proteomics” are relatively new words, coined by Wilkins et al. in 1996 [16]. The proteome is the entire set of proteins expressed by the genome. Proteomic analysis means a comprehensive analysis of proteins, and proteomics is the science by which proteins are comprehensively investigated with regard to their roles as functional elements. Recently, characterization of these cellular proteins by proteomic approaches has revealed that the surface charge of biomaterials defines the protein reactivity and the protein-biomaterial interaction. In the previous studies, several reports utilized proteomic approaches to explain the biomaterials-cells interaction. Titanium (Ti) is used commonly in implants and biomaterials. The surface modification was grafted by poly(sodium styrene sulfonate) (poly NaSS). The mechanisms of titanium alloy inducing platelet activation, that causes cell adsorption and proliferation, were identified by using two-dimensional gel electrophoresis (2-DE) combined with mass spectrometry, which may be related to protein adsorption on biomaterial surfaces [17]. Nanomaterials may release trace substances, which may be toxic to the surrounding cells. Human lung epithelial cells and human monocyte-derived macrophages were used to examine the cellular uptake of several forms of titanium dioxide nanoparticles and carbon nanotubes by using proteomic approaches [18]. The direct analysis of extracellular matrix (ECM) proteins from vascular aortic smooth muscle cells using a Protein Chip Bioprocessor and combined with surface enhanced laser desorption ionization time-of-flight mass spectrometry (SELDI-TOF MS) was developed by Lavigne and coworkers [19]. Their method involved a protein chip to analyze ECM proteins without transferring.

To evaluate the responses of fibroblasts to a CNTs/SF polymer surface, a quartz crystal microbalance (QCM) technique was used to quantify the mass of adhesion cells and immunochemical stains to observe the morphological changes of the cells. To apply proteomic approaches to develop a new tool for characterization of the responses of cells to biomaterials, a mass spectrometry-based profiling system was adopted. This system was able to assess characteristic proteins that were expressed due to the interactions of fibroblasts with biopolymer surfaces. Through the investigation, proteins that influence the responses and later proliferations of fibroblasts on biopolymer surfaces were identified, and CD44 was found to be involved in cell adhesion when SF interactions regulate signaling pathways.

## 2. Materials and Methods

### 2.1. Fabricating CNTs and Dispersing CNTs on the Electrode of QCM

The multiwalled carbon nanotubes (CNTs, 6–13 nm outer diameter, 2.5–20 *μ*m long) were treated by refluxing in concentrated nitric acid at 85°C for 3 h. When the CNTs were precipitated from the solution, the nitric acid was carefully removed. The mixture was then filtered through a 0.22 *μ*m filter under a vacuum condition. The CNTs were rinsed with D.I. water, collected, and dried in an oven at 50°C. The surface of a 9 MHz QCM gold electrode (ANT Tech, Taiwan) was washed with 1 M HCl, rinsed with D.I. water, and dried at room temperature. The frequency of the electrode measured by the QCM (ADS, ANT Tech, Taiwan) was assigned as *F*_0_ at the flow rate of 60 *μ*L/min of phosphate buffered saline (PBS). To prepare an electrode with CNTs decoration, a solution of pluronic F68 was applied to disperse CNTs on the electrode surface. Briefly, a 1% F68 solution was dropped onto the QCM gold electrode and then dried under oscillation. The CNTs in solution were then deposited onto the surface of the electrode and dried for further applications.

### 2.2. Atomic Force Microscopy Image of QCM Chip Surface

The QCM chip surfaces were analyzed by atomic force microscopy (AFM). The AFM images were acquired with a Slover PRO (NT-MDT, Russia) atomic force microscopy under ambient pressure. The semicontact mode was used with a frequency of 0.5 *μ*m/s to scan an area of 50 × 50 *μ*m^2^. The AFM probe was a golden silicon probe (NSG11, NT-MDT, Russia) with the length, width, thickness, resonant frequency, and force constant as 100 mm, 35 *μ*m, 2.0 *μ*m, 255 kHz, and 11.5 N/m^2^, respectively.

### 2.3. Adsorption of SF onto CNTs Polymer Surfaces Determined by QCM Measurements and Characterized by FT-IR

Silk cocoons were purchased from a silk center in Taiwan (ShihTan, Miao-Li, Taiwan). Briefly, silk cocoons were boiled in Na_2_CO_3_ and extracted SFs were then dissolved in 9.3 M LiBr solution. The final concentration of the SF aqueous solution was 8% (w/v). This concentration was determined by weighing the residual solid in a known solution volume after drying at 60°C. For fabricating a CNTs/SF electrode, 1% of the SF solution was injected into the flow loop with a CNTs dispersing electrode at the flow rate of 60 *μ*L/min. Moreover, double injections of SF solution were performed to assure that the adsorption of SF on the electrode was saturated. The frequency shifts (Δ*F*) were determined by the QCM and the masses of SF adsorption were recorded and calculated. To determine that the CNTs/SF layers were stably coated onto the electrode, the frequency of the electrode was measured during the flow of PBS for several minutes. The surface characterizations of the electrode decorated with CNTs, CNTs/SF were also observed using a Fourier transform infrared spectrometer (FT-IR, Spectrum One system, PerkinElmer, USA).

### 2.4. Culturing Fibroblasts on the CNTs and CNTs/SF Electrode Surfaces

The fibroblasts were maintained at 37°C and 5% CO_2_ in Dulbecco's Modified Eagle Medium (DMEM) supplemented with 10% fetal bovine serum (FBS, Hyclone Laboratories, Logan, UT), 1% penicillin/streptomycin (Gibco, Grand Island, NY, USA), and 44 mM NaHCO_3_.

Before seeding fibroblasts, the electrodes were sterilized with 70% (v/v) ethanol and then exposed under ultraviolet light for 30 min. Serum-free medium containing 4 × 10^4^ fibroblasts was added to each well in the presence of the aforementioned electrodes, and cells were incubated at 37°C with 5% CO_2_ for 12 h for investigating the adhesion of the cells on those electrodes [20]. After incubation, the electrodes were rinsed with PBS, and then frequency shifts were measured by the QCM.

### 2.5. BrdU Assay

The viability of the adhered cells was determined by BrdU assay (BrdU Cell Proliferation Assay, Millipore, USA). The assay was performed according to the manufacturer's instructions. Briefly, fibroblasts were seeded into a sterile 96-well tissue culture plate with a density of 2 × 10^5^ cells/mL in 100 *μ*L/well of appropriate cell culture media and incubated for 72 and 120 h. Then, cells were incubated in the medium containing BrdU reagent for 2 h. Fixing solution was added before the absorbencies were measured at 520 nm using an ELISA reader (Multiskan EX, Thermo Scientific, Vantaa, Finland, reference wavelength: 450 nm).

### 2.6. Proteomic Analysis of Fibroblasts on Various Surfaces

After incubation on different material surfaces, the fibroblasts were lysed by cell lysis buffer (3500-1, Epitomics, Inc., USA), and cell lysates were centrifuged at 1500 ×g for 10 min at 4°C. The supernatants were filtered by 0.8 *μ*m filters. The protein concentrations of the cell lysate samples were measured using a fluorescence-based protein quantification detection kit (Quant-iT Fluorometer, Qubit Protein Assay Kit, Q33212, Invitrogen), and the protein concentrations were adjusted to 1 mg/mL by 25 mM ammonium bicarbonate.

Cell lysate samples (100 *μ*L) were transferred into 1.5 mL Eppendorf tubes and incubated at 37°C for 3 h after mixing with 25 *μ*L of 1 M dithiothreitol (DTT, USB Corporation, 15397). Then the cell lysate samples were reduced and alkylated in the dark at room temperature for 30 min after the addition of 25 *μ*L of 1 M iodoacetamide (IAA, Amersham Biosciences, RPN6302V) in 25 mM ammonium bicarbonate. Approximately 10 *μ*L of 0.1 *μ*g/*μ*L modified trypsin digestion buffer (Trypsin Gold, Mass Spectrometry Grade, V5280, Promega, WI, USA) in 25 mM ammonium bicarbonate was added to the cell lysate samples, which were then incubated at 37°C for at least 12 h in a water bath. Two microliters of formic acid was added to each sample before mass spectrometric analysis for protein identification.

The complex peptide mixtures were separated by RP-nano-UPLC-ESI-MS/MS. The protein tryptic digests were fractionated using a flow rate of 400 nL/min with a nano-UPLC system (nanoACQUITY UPLC, Waters, Milford, MA) coupled to an ion trap mass spectrometer (LTQ Orbitrap Discovery Hybrid FTMS, Thermo, San Jose, CA) equipped with an electrospray ionization source. For RP-nano-UPLC-ESI-MS/MS analyses, a sample (2 *μ*L) of the desired peptide digest was loaded into the reverse phase column (Symmetry C18, 5 *μ*m, 180 *μ*m × 20 mm) by an autosampler. The RP separation was performed using a linear acetonitrile gradient from 99% buffer A (100% D.I. water/0.1% formic acid) to 85% buffer B (100% acetonitrile/0.1% formic acid) in 100 min using the micropump at a flow rate of approximately 400 nL/min. The separation was performed on a C18 microcapillary column (BEH C18, 1.7 *μ*m, 75 *μ*m × 100 mm) using the nanoseparation system. As peptides were eluted from the microcapillary column, they were electrosprayed into the ESI-MS/MS with the application of a distal 2.1 kV spraying voltage with heated capillary temperature of 200°C. Each cycle of one full-scan mass spectrum (*m*/*z* 400–2000) was followed by three data dependent tandem mass spectra with collision energy set at 35%.

### 2.7. Database Search

For protein identification, Mascot software (Version 2.2.1, Matrix Science, London, UK) was used to search the Swiss-Prot human protein sequence database. For proteolytic cleavages, only tryptic cleavage was allowed, and the number of maximal internal (missed) cleavage sites was set to 2. Variable modifications of cysteine with carboxyamidomethylation, methionine with oxidation, and asparagine/glutamine with deamidation were allowed. Mass tolerances of the precursor peptide ion and fragment ion were set to 10 ppm and 0.5 Da, respectively. When the Mowse score was greater than 30, the protein identification was defined as positive and considered significant (*P* < 0.05). Proteins were initially annotated by similar search conditions using UniProtKB/Swiss-Prot databases.

### 2.8. Western Blotting of Protein Expression

Confirmation of protein expression was performed by Western blotting. Each cell lysate sample (1 *μ*g/*μ*L, 10 *μ*L) was electrophoresed through a precast gel (NuPAGE Novex 4–12% Bis-Tris Gel, 1.5 mm, 10 wells, Invitrogen, Carlsbad, CA). Proteins were transferred from the gel to a polyvinyldifluoride (PVDF) membrane (Millipore, Bedford, CA) by means of the semidry technique using the Criterion Blotter (Bio-Rad) at 100 V for 60 min and blocked with 5% milk in PBS (adjusted to pH 7.4) containing 0.05% Tween 20. The membrane was then incubated overnight with primary rabbit antibody (1 *μ*g/mL) of anti-CD44 (1998-1, Epitomics, Inc.). After washing, the membrane was incubated with alkaline peroxidase-conjugated AffiniPure goat anti-rabbit IgG (111-035-003, Immuno Research) for 1 h (1 : 10000). Proteins were detected with an enhanced chemiluminescent (ECL) system, and quantitative analysis of Western blotting was carried out using the ImageQuant-TL-7.0 software, version 2010 (Amersham Biosciences).

### 2.9. Cell Morphology Observed by Immunochemical Staining

For cell morphology of adhered fibroblasts on the aforementioned electrodes after incubation, the electrodes were washed and fixed with 4% formaldehyde at 4°C. The nuclei and cytoskeleton of the cells were stained with 4′-6-diamidino-2-phenylindole (DAPI, 32670, Sigma-Aldrich, USA) and vimentin (Vimentin DyLight 488 Antibody, Epitomics, USA), respectively. In addition to staining with DAPI and vimentin, the anti-CD44 antibody (1998-1, Epitomics, Inc) was incubated and followed by staining with Alexa Fluor 568 goat anti-rabbit IgG (A-11011, Invitrogen). The samples were blocked with 2% bovine serum albumin (BSA, A1933, Sigma-Aldrich, USA) at room temperature for 30 min. The cell images were observed by a microscope equipped with fluorescence light source (FLoid Cell Fluorescence Imaging Station, Invitrogen), and the cell micrographs were taken with a CCD camera.

### 2.10. Statistical Analysis

All calculations used the SigmaStat statistical software (Jandel Science Corp., San Rafael, CA). All statistical significances were evaluated at 95% of confidence level or better. Data are presented as mean ± standard error.

## 3. Results and Discussion

### 3.1. Characterizations of Electrodes of QCM Decorated with CNTs and CNTs/SF

**Figure 1:**
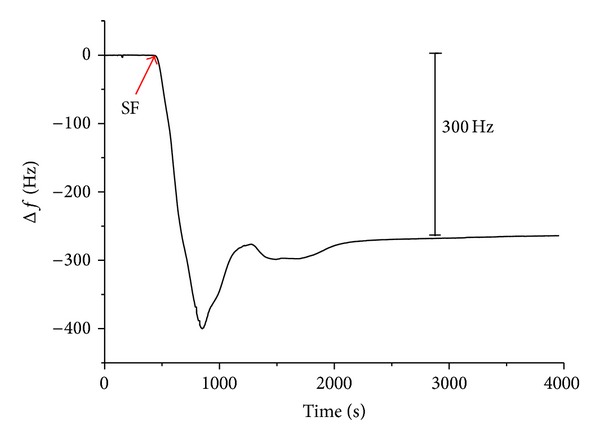
A representative of the frequency shift for preparing the electrodes decorated with CNTs/SF layer. The frequency shift of CNTs/SF exhibited the least frequency response around −335 ± 21 Hz, *n* = 7.

To prepare CNTs/SF layer, SF was adsorbed onto a CNTs electrode surface using the layer-by-layer technique [21, 22]. For each tested biopolymer, the frequency shifts dropped sharply, as it was absorbed onto the electrode surface (Figure 1). The theory for QCM detections can be described by the Sauerbrey equation, Sauerbrey equation in gas phase. Δ*F* is the frequency shift (Hz); *F* is basic oscillation frequency of piezoelectric quartz (Hz); *A* is the active area of QCM (cm^2^); Δ*M* is the mass change on QCM (g). Consider the following:

(1) $$\begin{array}{c} \Delta F=-2.3\times 10^{-6}\frac{F^{2}\Delta M}{A}, \end{array}$$

which gives the mass change as proportional to the shift in the oscillation frequency of the piezoelectric quartz crystal [20]. QCMs with electrodes have been widely studied in several fields such as environmental protection, medicine, and biotechnology. Additionally, monitoring biomolecular interactions in immunology and investigating cell-substrate communications have been extensively studied [6, 7]. Recently, modifications of electrodes with various biopolymers of QCM have been used to detect the adhesion of cells [20]. The QCM frequency variation after CNTs-biopolymer formation was lowered to around 2.3 kHz.  Table 1 presents the frequency responses and mass to the absorption of CNTs and CNTs/SF by using the Sauerbrey equation [23]. CNTs exhibited the strongest frequency responses upon deposition on the electrode (−2004 ± 33 Hz, 1377 ± 23 ng, *n* = 7) while CNTs/SF exhibited the least frequency response (−335 ± 21 Hz, 231 ± 30 ng, *n* = 7).

**Figure 2:**
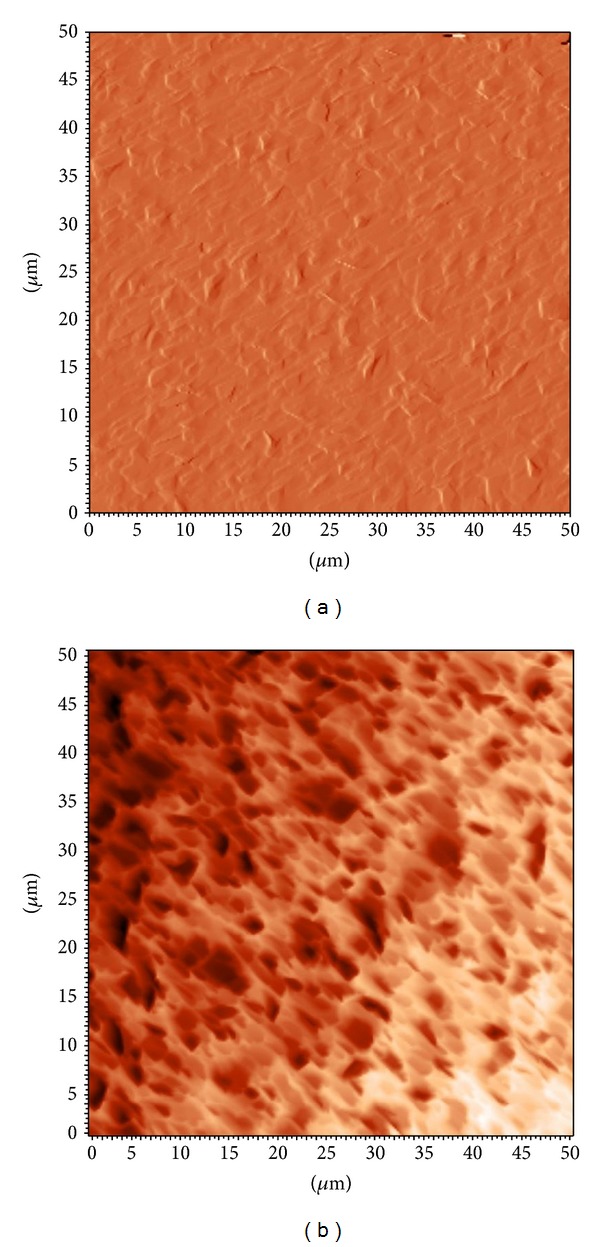
AFM images of the QCM chip. (a) Blank, 50 × 50 *μ*m, (b) CNTs, 50 × 50 *μ*m. AFM measurements could also be used for measuring the surface roughness of the QCM chip. The mean surface roughness was 1.0 and 2.3 nm for blank and CNTs surfaces, respectively.

To investigate the topology characteristics of the surface, AFM was used to observe the QCM chip surface. In Figure 2, the image of the topographical map taken in the semicontact mode of a 50 × 50 *μ*m^2^ zone is shown.  Figure 2(a) is a surface image of the QCM chip, and Figure 2(b) shows the CNTs surface. This impressive image in Figure 2(b) shows the surface roughness with a mean depth of about 2.3 *μ*m. Certainly, a rough surface may provide the opportunity to increase the reaction surface and the effectiveness of cell adhesion.

**Figure 3:**
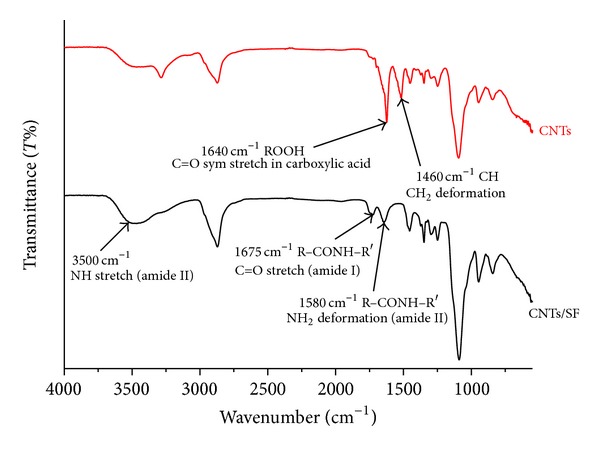
The ATR-FTIR transmission spectra of the CNTs and CNTs/SF layers decorated on electrodes of QCM. The peaks at 1580 cm^−1^ and 1675 cm^−1^ in the spectra were attributed to amide II and amide I, confirming the presence of amide I and II in SF modified surface.

Modified surfaces of electrodes of QCM were also routinely characterized using FT-IR spectrum.  Figure 3 displays the characteristics of the FT-IR spectra of the aforementioned polymers. In the absorption curve of CNTs, the broad band at 3400 cm^−1^ was attributed to the OH functional group from F68, a dispersing agent for CNTs, owing to its polyethylene oxide- (PEO-) polypropylene oxide structure [4]. The absorption bands at 1640 and 1460 cm^−1^ were assigned to C=O stretching and CH_2_ deformation in carboxylic acid, which were attributed to the acid treatment of the CNTs [24]. The absorption band at 3500 cm^−1^ in the second curve corresponds to a NH stretch of SF. The peaks at 1580 cm^−1^ and 1675 cm^−1^ in the spectra of the SF surface were attributed to amide II (R–NHR′, NH_2_ deformation, N–H bending, and C–N stretching) and amide I (R–CONHR′, C=O stretching), respectively, confirming the presence of amide I and II in SF [25]. These results indicate the presence of O=C–NH species, which are derived from carboxylic acid and amide structures. Accordingly, the polycomplex between CNTs and SF was formed when amino groups in SF formed complexes with carboxyl groups in CNTs [26]. The spectra indicated that the electrodes were successfully decorated with CNTs and CNTs/SF biopolymers.

### 3.2. Quantitative Analysis of Fibroblasts Adhesion on Electrodes

To investigate the adhesion of fibroblasts onto electrodes decorated by CNTs and CNTs/SF polymer surfaces, fibroblasts were incubated on the electrodes for 12 h. Since the adsorption of various proteins of bovine serum onto the aforementioned surfaces may influence cell adsorption behaviors, a serum-free medium was used in the cell culture. The cultivation of fibroblasts under serum-free conditions for 12 h herein prevented the apoptosis and proliferation of cells [27]. The results concerning the adhesion of fibroblasts onto the electrode of QCM that was decorated by CNTs or CNTs/SF were obtained from the frequency shifts [20]. The frequency shifts for nonmodified surfaces, CNTs-coated electrodes, and CNTs/SF-coated electrodes were −16.05 ± 0.44, −24.85 ± 0.30, and −29.43 ± 0.77 × 10^3^ Hz; the attached cell masses corresponding to those surfaces were 11.02 ± 0.30, 17.07 ± 0.21, and 21.52 ± 0.49 × 10^3^ ng (Table 2, *P* < 0.001, *n* = 10), respectively. The amount of fibroblasts that adhered to the CNTs/SF-coated electrode significantly exceeded that coated with either of the other surfaces. The mass of the fibroblasts that adhered to the CNTs/SF-coated electrode was calculated markedly to exceed that of those that adhered to the other surfaces, such as the CNTs polymer surface. In this investigation, the results obtained using the QCM technique to examine the adhesion of fibroblasts to the polymer-coated surfaces of the electrodes were consistent with others [20].

### 3.3. BrdU Cell Proliferation Assay

**Figure 4:**
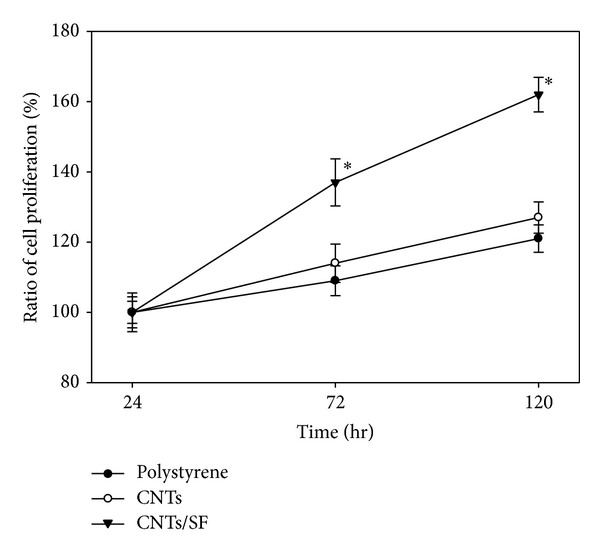
Proliferation (BrdU) test of fibroblasts on surfaces of polystyrene, CNTs and CNTs/SF. (polystyrene served as a control, *n* = 10, mean ± standard error, *P < 0.05,* t*-test).

The surface modifications were adopted to evaluate cell viability by BrdU cell proliferation assay. The BrdU cell proliferation assay is an artificial nucleoside that is an analogue of thymidine and used to detect* in vitro* cell proliferation rates [28, 29].  Figure 4 presents the result of the BrdU cell proliferation assay. On day one, no significant difference existed between the aforementioned nonmodified (polystyrene) and CNTs polymer surfaces, but the difference between the CNTs/SF and CNTs polymer surfaces was significant (*P* < 0.05). The number of adherent cells on the CNTs/SF polymer surface was 1.22 times that on the CNTs polymer surface (OD intensity: CNTs polymer surface, 0.0669; CNTs/SF polymer surface, 0.0816). This result of the BrdU assay is consistent with the shifts in the frequency of the QCM (different by a factor of 1.26-fold, Table 2, Δ*m*). Since the number of absorbed cells may vary among plates, normalization is required. Therefore, data after three and five days were compared with those after one day. On day three, the amount of fibroblasts that adhered to the CNTs/SF polymer surface notably exceeded the numbers on the other surfaces. Almost 37% more cells were present on CNTs/SF polymer surface than on the original nonmodified surface, while only 9% of cells were increased on the nonmodified surface, and no significant difference was observed between the CNTs polymer surface and the nonmodified surface. On the fifth day, regardless of whether the number of cells had greatly increased, the percentage difference between the number of newly synthesized cells on the nonmodified surface and that on the CNTs polymer surface was the same as that on the third day (6%). Nevertheless, the difference between the number of cells on the CNTs/SF polymer surface and that on the nonmodified surface had increased from 28 to 41%. Hence, the results demonstrate that cells on the CNTs and nonmodified surface grew at similar rates while those on the CNTs/SF polymer surface grew more rapidly. These results provided the evidence that SF accelerated adult cell proliferation.

### 3.4. Results of Proteomic Analysis

**Figure 5:**
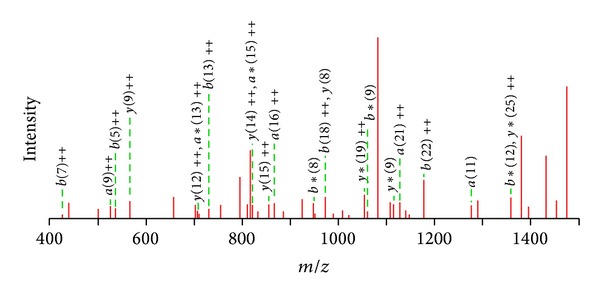
MS/MS spectrum of peptide from the fibroblasts incubated on CNTs/SF polymer surface. The amino acid sequence of the tryptic peptide is R.TPQIPEWLIILASLLALALILAVCIAVNSRRR.C (*m/z* = 1196.02, +3, from CD44). Interpretation of the complete *y*-ion and *b*-ion series provides the peptide sequences as shown.

To investigate the effect of CNTs/SF polymer surface on fibroblasts, a proteomic approach, such as RP-nano-UPLC-ESI-MS/MS analysis, was utilized to analyze cell lysates. The traditional method uses individual antibodies to evaluate the response of a cell to a surface, but the proteomic approach can be used to analyze an enormous number of proteins simultaneously. In this study, fibroblasts were incubated on various modified surfaces with serum-free medium. After 12 h, the cells were lysed, and the cell lysates were digested by trypsin, generating tryptic peptides that were subsequently analyzed by RP-nano-UPLC-ESI-MS/MS. The RP-nano-UPLC-ESI-MS/MS approach is perhaps the most representative method in proteome research. The fragmentation spectra obtained by the RP-nano-UPLC-ESI-MS/MS analysis in gradient detection mode were compared with a nonredundant protein database using Mascot software. When a protein was identified by three or more unique peptides, no visual assessment of spectra was conducted and the protein was considered to be present in the sample.  Figure 5 shows typical MS/MS spectrum of the identified peptides. The MS/MS spectrum represents the amino acid sequence of tryptic peptide, which is triply charged peptides with* m/z* of 1196.02. The amino acid sequence of the tryptic peptide is TPQIPEWLIILASLLALALILAVCIAVNSRRR. These peptides originated from CD44, and the interpretation of the complete*y*-ion and *b*-ion series provides the peptide sequence as shown.

The database search resulted in 127 proteins and most of these were identified at the minimal confidence level, which was only one unique peptide sequence matched. Experimental results reported a total of 17 protein identifications with higher confidence levels (Table 3, at least three unique peptide sequences matched), in which CD44 exhibited significant differences between the CNTs/SF and CNTs or nonmodified surfaces. CD44 was involved in cell differentiation, division, and cycle regulation, which was only found in the cell lysate samples from the CNTs/SF polymer surface and selected for validation by Western blot analysis and fluorescence image.

It has been well known that collagen plays important roles in cell adhesion progress [30–33]. In addition, other ECM proteins, such as lamina and fibronectin, were also involved in cell adhesion progress [33, 34]. The aim of this study was to develop a mass spectrometry-based analysis platform and to map the potential proteins and effective pathways associated with cell adsorption on a SF-surface. Thus, the influences of aforementioned proteins on cell adhesion were excluded in our study.  Table 4 shows the identified peptides and ontologies of CD44. Proteins were initially annotated by similarity searches using Swiss-Prot/TrEMBL and Bioinformatic Harvester EMBL databases; then, the known functions of the protein could be examined.

CD44 forms a ubiquitously expressed family of cell surface adhering molecules. It is a cell surface glycoprotein that participates in cell-cell and cell-matrix interactions, cell adhesion, and migration. The CD44 gene has only been detected in the higher levels of organisms and the amino acid sequence of the molecule is conserved among mammalian species. CD44 participates in adhesion and migration by binding to SF and other molecules in the ECM [35]. The main ligand of CD44 is hyaluronic acid (HA), an integral component of the ECM. Other CD44 ligands include osteopontin, serglycin, collagens, fibronectin, laminin, SF, and matrix metalloproteinases (MMPs) [36]. The CD44 transmembrane glycoprotein family adds new aspects to these roles by participating in signal transduction processes, which include the establishment of specific transmembrane complexes, and signaling a cascade organizer associated with the actin cytoskeleton [37]. CD44 may function as cellular growth factors, which may be important in tumor metastasis [38].

To validate the influence of CD44 for fibroblast adhesion on the CNTs/SF polymer surface, the cells were blocked by a CD44 antibody and the cell adhesion on the CNTs/SF polymer surface was measured by the QCM technique. When fibroblasts were preincubated with the CD44 antibody, the frequency shift was reduced (from −29.43 ± 0.77 × 10^3^ Hz to −23.64 ± 0.58 × 10^3^ Hz). The result of significantly decreasing the weight of the blocked fibroblasts adhering to CNTs/SF polymer surface was obtained. Through this experiment, CD44 was confirmed to play roles on the cell adhesion which may be associated with the cell adsorption pathway on cell-CNTs/SF polymer surface interactions.

**Figure 6:**
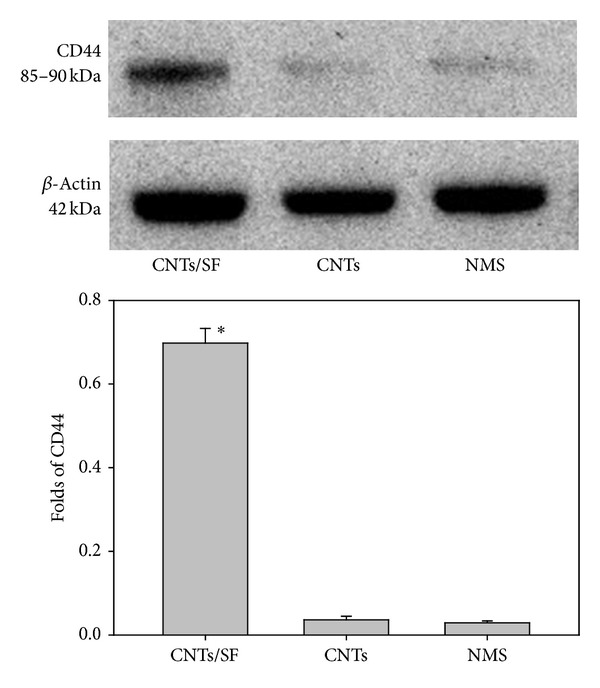
Immunoreactive bands of CD44 and *β*-actin from fibroblast cells cultured on surfaces of CNTs/SF, CNTs, and NMS (nonmodified surface). The quantitative analysis of Western blotting was carried out using the ImageQuant-TL-7.0 software. These values that refer to the expression of CD44 were normalized by the expression of beta-actin.

To confirm proteins identified by RP-nano-UPLC-ESI-MS/MS, Western blot analysis was applied to detect the candidate protein that may be associated with cell adhesion/growth pathways on the CNTs/SF polymer surface.  Figure 6 presents representative results of the Western blot analyses of cell lysates. CD44 was detected strongly in the cell lysates from the CNTs/SF polymer surface, which is valuable in confirming the SF-induced cell adhesion. In Figure 6, the *β*-actin was used as a marker for concentration normalization. Compared with the results of Western blotting, the concentration of CD44 in cell lysates from the CNTs/SF polymer surface was 23-fold more than those from nonmodified and CNTs polymer surfaces. This comparison was made using the quantitative analysis software ImageQuant-TL-7.0, and the *P* value was less than 0.05.

### 3.5. Cell Morphology by Fluorescence Microscopy

**Figure 7:**
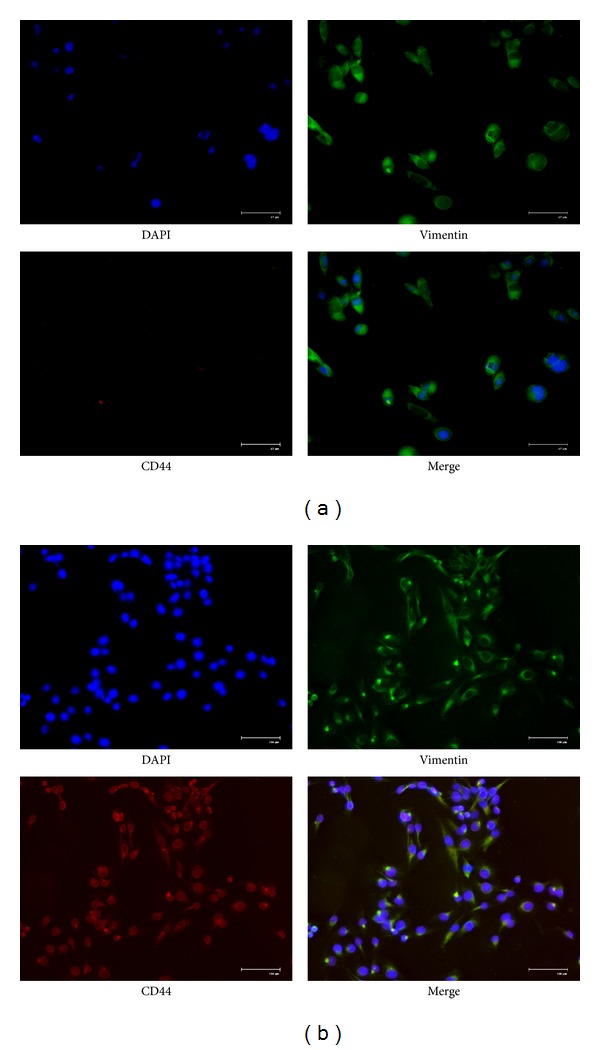
Immunochemical stains for DAPI (blue), vimentin (green), and CD44 (red) for adhered fibroblasts on CNTs and CNTs/SF polymer surfaces for 12 h of incubation to observe the morphology of the cells ((a) CNTs polymer surface; (b) CNTs/SF polymer surface; scale bar: 67 *μ*m for panel (a), 100 *μ*m for panel (b)).

**Figure 8:**
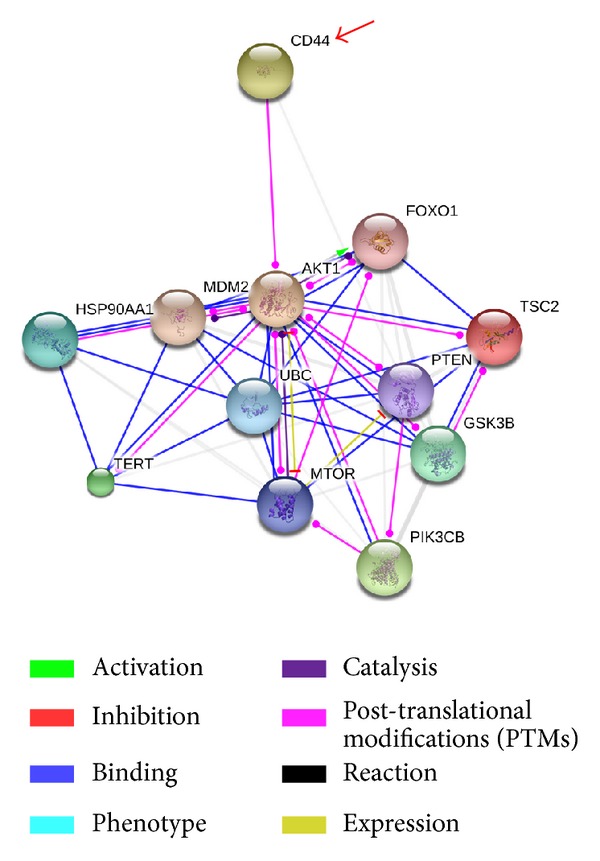
The CD44 protein-protein interaction pathways were performed by String 9.0 Web software. The CD44 can turn on the PI3 K/AKT/mTOR pathway, which is responsible for the proliferation and is required for survival of the majority of cells.

Fibroblasts were cultured in the medium with CD44 antibody onto CNTs/SF polymer surfaces. The cells were observed by immunochemical staining under fluorescence microscopes to determine the morphology of the adhering fibroblasts. In Figure 7 ((a) CNTs polymer surface; (b) CNTs/SF polymer surface; DAPI, blue; vimentin, green; CD44, red; 600X, scale bar: 67 *μ*m for panel A, 100 *μ*m for panel B), the cell fluorescence images showed that CD44 was present in the cell nucleus and membrane. In the present work, we show that the CD44 protein localizes to the nucleus and colocalizes with actin in the external side of plasma membrane protrusions. The cell images showed that the adopted antibodies successfully entered into cells and have the right localization. The CD44 protein-protein interaction pathways were performed by String 9.0 Web software (Figure 8). The CD44 can turn on the PI3 K/AKT/mTOR pathway, which is responsible for the proliferation and is required for survival of the majority of cells. The hypothesis of the mTOR pathway is that it acts as a master switch of cellular catabolism and anabolism, thereby determining the growth and proliferation of the cells. Activation of PI3 K/AKT/mTOR signaling through mutation of pathway components as well as through activation of upstream signaling molecules occurs in the majority of cells contributing to deregulation of proliferation, resistance to apoptosis, and changes in the metabolism characteristic of transforming cells.

As expected, the CNTs/SF polymer surface caused larger frequency shifts than the CNTs polymer surface. Indeed, the presence of SF on the surface supported* in vitro* cell adhesion, and SF participates importantly in cell proliferation [39]. In this study, the results were mutually consistent when using the BrdU cell proliferation assay and QCM techniques which were utilized to investigate the adhesions of fibroblasts to polymer surfaces that coated the electrodes. However, these methods are limited to the quantitative analysis of cell amount. Proteomic analysis provides a means for the large-scale characterization of the differential expression of proteins from cells on modified surfaces. The mass spectrometry-based proteomics approach has many advantages, especially in identification of related proteins. Experimental results indicate that the CNTs/SF polymer surface may be able to activate several cell-material interaction pathways and promote cell adhesion. Consequently, previous unfamiliar proteins can be found and the interaction of cell-material may be established. The proteomic scheme was adopted to identify proteins with differential expression, which participate importantly in the adhesion of cells to material surfaces.

## 4. Conclusions

In this study, a biopolymer surface was formatted with SF. According to the results concerning fibroblasts that were stained with DAPI/vimentin/CD44, BrdU cell proliferation assay, and the frequency shifts that were determined using QCM, the numbers and mass of fibroblasts that adhered to the CNTs/SF polymer surface of electrodes were significantly higher than those of fibroblasts on other surfaces. The SF modified surface has been confirmed and improved the cell adhesion. To evaluate the responses of cellular proteins induced by SF-modified surfaces, mass spectrometry-based proteomics is adopted to analyze complex proteins of cell lysate and to profile proteins based on their associated cell-surface interactions. By utilizing proteomic approaches, it is indicated that the SF modified surface induces fibroblasts to express CD44 as an interactive protein between cell and material surface to enhance cell adhesion. Although the pathways of the interactions between CD44 and SF were unclear, the cell adhesion affected by CD44 was established. In summary, the functional groups of biomaterials may induce the secretion of proteins from cells. This study proposed a new approach for the detection of proteins to assess the response of fibroblasts to a material surface. Knowing the responses of cellular proteins induced by biomaterials may assist the development of applications in the immediate future.

## Figures and Tables

**Table 1:** Frequency shifts and mass for the adsorption of CNTs and CNTs/SF layers measured by the QCM and calculated by the Sauerbrey equation.

Adsorption polymer	Δ*F* (Hz)	Δ*m* (ng)
CNTs	−2004 ± 33	1377 ± 23
CNTs/SF	−335 ± 21	231 ± 30

Data are expressed as mean ± standard error, *n* = 7.

**Table 2:** Frequency shifts of QCM and weights of adhered fibroblasts on the electrodes decorated with nonmodified surface, CNTs, and CNTs/SF layers for 12 h of cell incubation.

Cell adhesion	Δ*F* (×10^3^ Hz)	Δ*m* (×10^3^ ng)
Nonmodified surface	−16.05 ± 0.44	11.02 ± 0.30
CNTs	−24.85 ± 0.30*	17.07 ± 0.21*
CNTs/SF	−29.43 ± 0.77*	21.52 ± 0.49*

Data are expressed as mean ± standard error, *n* = 10, *P < 0.001 (*t*-test).

**Table 3:** The 17 proteins identified with higher confidence level (at least three unique peptide sequences matched) in this study. The P17, CD44, was only identified on the SF modified surface.

Protein number	Swiss-Prot/ TrEMBL accession number	Protein name	MW (Da)	Score	Match queries	PI	Sequence coverage	Match peptide
P01	Q2M3C7	A-kinase anchor protein SPHKAP	186339	34	3	5.04	8%	R.EACAGEPEPFLSK.S + Carbamidomethyl (C); Phospho (ST) R.QSSMPDSRSPCSR.L + Carbamidomethyl (C); 4 Phospho (ST); Oxidation (M) R.SPVCHRQSSMPDSRSPCSR.L + Carbamidomethyl (C); Deamidated (NQ); 4 Phospho (ST); Oxidation (M)

P02	P05067	Amyloid beta A4 protein	86888	19	3	4.73	9%	K.WDSDPSGTKTCIDTK.E + 5 Phospho (ST) K.GAIIGLMVGGVVIATVIVITLVMLKK.K + Oxidation (M); Phospho (ST) R.ALEVPTDGNAGLLAEPQIAMFCGR.L + 2 Deamidated (NQ); Oxidation (M); Phospho (ST)

P03	Q9UKV3	Apoptotic chromatin condensation inducer in the nucleus	151771	19	3	6.08	2%	R.EREMER.R R.TSTSSSSVQAR.R + 7 Phospho (ST) K.QSADSSSSRSSSSSSSSSR.S + Deamidated (NQ); 9 Phospho (ST)

P04	Q9NR09	Baculoviral IAP repeat-containing protein 6	529919	34	3	5.67	4%	R.SRGTPSGTQSSR.E + Deamidated (NQ); 3 Phospho (ST) R.TIPDKIGSTSGAEAANK.I + Deamidated (NQ) R.GRTIPDKIGSTSGAEAANK.I + Phospho (ST)

P05	P51685	C-C chemokine receptor type 8	40817	18	3	8.66	4%	R.ESCEKSSSCQQHSSR.S + Carbamidomethyl (C); Deamidated (NQ); 3 Phospho (ST) R.ESCEKSSSCQQHSSR.S + Carbamidomethyl (C); 2 Deamidated (NQ); 5 Phospho (ST) R.ESCEKSSSCQQHSSR.S + Carbamidomethyl (C); 2 Deamidated (NQ); 5 Phospho (ST)

P06	Q9BV73	Centrosome-associated protein CEP250	280967	19	3	5	5%	R.EPAQLLLLLAK.T K.GQLEVQIQTVTQAK.E + 4 Deamidated (NQ); Phospho (ST) K.AEHVRLSGSLLTCCLRLTVGAQSR.E R.SLFKRGPLLTALSAEAVASALHK.L + 3 Phospho (ST)

P07	O95067	G2/mitotic-specific cyclin-B2	45253	18	3	9	12%	R.KKLQLVGITALLLASK.Y K.VPVQPTKTTNVNKQLKPTASVKPVQMEK.L + Deamidated (NQ); Oxidation (M); Phospho (ST) K.AQNTKVPVQPTKTTNVNK.Q + 3 Deamidated (NQ); 2 Phospho (ST)

P08	Q16478	Glutamate receptor, ionotropic kainate 5	109195	42	4	8.54	7%	K.VSTIIIDANASISHLILRK.A + Deamidated (NQ); 2 Phospho (ST) R.LNCNLTQIGGLLDTK.G + 2 Deamidated (NQ); Phospho (ST) R.YQTYQRMWNYMQSK.Q + 4 Deamidated (NQ); Oxidation (M); 2 Phospho (ST); 2 Phospho (Y) R.LQYLRFASVSLYPSNEDVSLAVSRILK.S + 2 Deamidated (NQ)

P09	Q63HM2	Pecanex-like protein C14orf135	132616	36	7	5.88	9%	K.GDLIKVLVWILVQYCSK.R K.GDLIKVLVWILVQYCSK.R + Deamidated (NQ) K.HQLKDLPGTNLFIPGSVESQR.V K.HQLKDLPGTNLFIPGSVESQR.V + Deamidated (NQ) K.HQLKDLPGTNLFIPGSVESQR.V + 2 Deamidated (NQ) R.LMWIMILECGYTYCSINIK.G + 2 Carbamidomethyl (C); Deamidated (NQ) K.KYVANTVFHSILAGLACGLGTWYLLPNR.I + Carbamidomethyl (C)

P10	O14497	AT-rich interactive domain-containing protein 1A	241892	27	8	6.24	6%	K.SKKSSSSTTTNEK.I + Deamidated (NQ); 6 Phospho (ST) K.HPGLLLILGKLILLHHK.H R.NSMTPNPGYQPSMNTSDMMGR.M + 2 Deamidated (NQ); Oxidation (M); 2 Phospho (ST) R.EMAVVLLANLAQGDSLAARAIAVQK.G + Deamidated (NQ); Oxidation (M) R.ITATMDDMLSTRSSTLTEDGAK.S + 2 Oxidation (M); 4 Phospho (ST) K.APGSDPFMSSGQGPNGGMGDPYSR.A + 4 Phospho (ST); Phospho (Y) R.GYMQRNPQMPQYSSPQPGSALSPR.Q + Oxidation (M); Phospho (ST) K.RNSMTPNPGYQPSMNTSDMMGR.M + Deamidated (NQ); Oxidation (M); 3 Phospho (ST); Phospho (Y)

P11	P50224	Sulfotransferase 1A3/1A4	34174	23	4	5.68	6%	R.LIKSHLPLALLPQTLLDQK.V R.LIKSHLPLALLPQTLLDQK.V + Deamidated (NQ) R.LIKSHLPLALLPQTLLDQK.V + Deamidated (NQ) R.LIKSHLPLALLPQTLLDQK.V + 2 Deamidated (NQ)

P12	Q09666	Neuroblast differentiation-associated protein AHNAK	628699	39	3	5.8	2%	K.VHAPGLNLSGVGGKMQVGGDGVK.V + Deamidated (NQ); Oxidation (M); Phospho (ST) R.AGAISASGPELQGAGHSKLQVTMPGIKVGGSGVNVNAK.G + 2 Deamidated (NQ); Oxidation (M); 4 Phospho (ST) K.VKVPEVDVRGPK.V

P13	Q63HM2	Pecanex-like protein C14orf135	132616	35	3	5.88	5%	R.TSCMPSSKMK.E + Carbamidomethyl (C); 2 Oxidation (M); 2 Phospho (ST) K.HQLKDLPGTNLFIPGSVESQR.V + Deamidated (NQ) K.HQLKDLPGTNLFIPGSVESQR.V + 2 Deamidated (NQ)

P14	O43182	Rho GTPase-activating protein 6	105882	32	4	7	9%	R.EQQVTQK.K K.DPGMTGSSGDIFESSSLR.A + Phospho (ST) -.MSAQSLLHSVFSCSSPASSSAASAK.G + Deamidated (NQ); Oxidation (M); 3 Phospho (ST) R.EQQVTQKKLSSANSLPAGEQDSPR.L + 2 Phospho (ST)

P15	O76074	cGMP-specific 3′, 5′-cyclic phosphodiesterase	99921	39	5	5.74	10%	R.WILSVKKNYR.K + Phospho (ST) K.KIAATIISFMQVQK.C + Oxidation (M); Phospho (ST) K.ELNIEPTDLMNREKK.N + Deamidated (NQ) K.TQSILCMPIKNHREEVVGVAQAINK.K + 4 Deamidated (NQ); 2 Phospho (ST) R.GHTESCSCPLQQSPRADNSAPGTPTRK.I + 2 Deamidated (NQ); Phospho (ST)

P16	O60299	ProSAP-interacting protein 1	71747	35	5	7.56	9%	K.SRTMTPAGGSGSGLSDSGR.N + Oxidation (M) K.SRTMTPAGGSGSGLSDSGR.N + Oxidation (M); 4 Phospho (ST) R.IGTASYGSGSGGSSGGGSGYQDLGTSDSGR.A + 4 Phospho (ST); Phospho (Y) K.SRTMTPAGGSGSGLSDSGR.N + Oxidation (M); 4 Phospho (ST) K.QLQLSYVEMYQRNQQLER.R + 3 Deamidated (NQ); Phospho (Y)

P17	P16070	CD44	81487	79	3	5.13	16%	R.YGFIEGHVVIPR.I R.TPQIPEWLIILASLLALALILAVCIAVNSRRR.C K.SQEMVHLVNKESSETPDQFMTADETRNLQNVDMK.I

**Table 4:** The identified peptides and gene ontologies of CD44.

Accession number	Protein name	Subcellular location	Biological process	Molecular function
P16070	CD44	Membrane, cytoplasm, Golgi apparatus	Cell adhesion, cellular response to fibroblast growth factor stimulus	Blood group antigen, receptor, collagen binding

CD44 is the receptor for hyaluronic acid (HA) and mediates cell-cell and cell-matrix interactions through its affinity for HA and possibly also through its affinity for other ligands such as osteopontin, collagens, and matrix metalloproteinases (MMPs).

## Novelty of the Study

The preparation and characterization of silk fibroin modified surface were confined. The pathway of silk fibroin biopolymer surface induced cell membrane protein activation was identified by proteomic approaches. The silk fibroin biopolymer surface may induce and activate CD44 to enhance cell adhesion and proliferation.
